# Latency-Associated Nuclear Antigen (LANA) Promotes Ferroptosis by Suppressing Nrf2/GPX4 and Upregulating MDM2

**DOI:** 10.3390/pathogens14060590

**Published:** 2025-06-15

**Authors:** Yuejia Cao, Shihan Shao, Yingying Zhang, Dandan Song, Fei Gui, Xinyi Chen, Yu Hong, Rong Chen, Yang Song, Dongmei Li, Xiaohua Tan, Chunhong Di

**Affiliations:** 1 School of Public Health, Hangzhou Normal University, Hangzhou 311121, China; 2023111024002@stu.hznu.edu.cn (Y.C.); shaoshihan08@163.com (S.S.); 2022112027019@stu.hznu.edu.cn (Y.Z.); 20207059@hznu.edu.cn (D.S.); 20197077@hznu.edu.cn (F.G.); 2024112024006@stu.hznu.edu.cn (X.C.); hongyu@hznu.edu.cn (Y.H.); rchen1984@163.com (R.C.); 20160042@hznu.edu.cn (Y.S.); 2 The Affiliated Hospital, Hangzhou Normal University, Hangzhou 310015, China; 3 School of Medicine, Shihezi University, Shihezi 832002, China; lidong_abc@126.com

**Keywords:** Kaposi’s sarcoma-associated herpesvirus (KSHV), latency-associated nuclear antigen (LANA), ferroptosis, nuclear factor erythroid 2-related factor 2 (Nrf2), mouse-double minute 2 (MDM2)

## Abstract

Ferroptosis, an iron-dependent cell death driven by lipid peroxidation, is regulated by key mediators including glutathione peroxidase 4 (GPX4) and nuclear factor erythroid 2-related factor 2 (Nrf2). Kaposi’s sarcoma-associated herpesvirus (KSHV) encodes latency-associated nuclear antigen (LANA), a multifunctional protein critical for viral persistence. Although studies reported that KSHV infection enhanced cellular resistance to ferroptosis, the specific role of LANA in this process remains unexplored. Here, we demonstrate that LANA unexpectedly promotes ferroptosis. In KSHV-positive iSLK.219 cells, LANA knockdown significantly attenuated RSL-3-induced ferroptosis, whereas LANA overexpression sensitized HeLa cells to ferroptotic death. Quantitative analysis revealed that LANA-depleted cells exhibited significantly elevated ROS accumulation (*p* < 0.01), whereas LANA-overexpressing cells maintained reduced ROS levels during challenge with the ferroptosis inducer RSl-3. Mechanistically, LANA suppressed glutathione peroxidase 4 (GPX4) expression, reduced nuclear factor erythroid 2-related factor 2 (Nrf2) expression and impaired its nuclear translocation, and upregulated mouse double minute 2 homolog (MDM2) expression. Pharmacological inhibition of Nrf2 (ML385) or MDM2 (nutlin3a) reversed the ferroptotic effects of LANA knockdown or overexpression, respectively. These findings reveal a pro-ferroptotic role of LANA via Nrf2/GPX4 suppression and MDM2 activation.

## 1. Introduction

Kaposi’s sarcoma-associated herpesvirus (KSHV, human herpesvirus 8) is recognized as an oncogenic γ-herpesvirus associated with Kaposi sarcoma (KS), multicentric Castleman disease (MCD), and primary effusion lymphoma (PEL) [1,2]. Similar to other herpesviruses, KSHV exhibits a biphasic life cycle alternating between latent and lytic phases, with both phases contributing to disease pathogenesis [3,4]. During latency, the virus expresses a limited set of genes, including the latency-associated nuclear antigen (LANA), viral cyclin (vCYC), and viral FLICE inhibitory protein (vFLIP). Among these, LANA plays a pivotal role in establishing and maintaining viral latency [5,6].

Regulated cell death (RCD) is defined as an essential biological process for eliminating damaged cells and maintaining tissue homeostasis. Ferroptosis, first characterized in 2012 [7], represents a novel iron-dependent form of RCD driven by lipid peroxidation. Glutathione peroxidase 4 (GPX4) serves as the primary ferroptosis suppressor, utilizing glutathione (GSH) to reduce lipid peroxides into non-toxic alcohols, thereby preventing lethal lipid peroxide accumulation. Cellular susceptibility to ferroptosis increases with GPX4 inhibition or reduced expression levels, while GPX4 overexpression confers significant protection against ferroptosis. Nuclear factor erythroid 2-related factor 2 (Nrf2), a master regulator of antioxidant responses, suppresses ferroptosis by upregulating antioxidant enzymes including GPX4 [8].

The tumor suppressor protein p53 plays a crucial role in the induction of apoptosis. The murine double minute 2 homolog (MDM2) directly interacts with p53, thereby negatively regulating its activity [9]. Although MDM2 functions to inhibit p53-induced apoptosis, it promotes ferroptosis [10]. LANA inhibits the interaction between MDM2 and P53 and suppresses apoptosis [11].

Although studies reported that KSHV infection enhanced cellular resistance to ferroptosis, the specific role of LANA in this process remains unexplored. Previously, studies identified direct interaction between LANA and Nrf2; however, the impact of LANA on Nrf2 expression levels has not been elucidated. Furthermore, while LANA modulates the MDM2–p53 interaction [12], its regulatory effect on MDM2 expression remains undefined.

In this study, we systematically investigated the functional relationship between LANA and ferroptosis. Through RNA interference-mediated LANA knockdown in KSHV latently infected iSLK.219 cells and ectopic expression LANA in KSHV-negative HeLa cells, combined with ferroptosis inducer RSL-3 treatment, we unexpectedly observed that LANA potentiated ferroptotic sensitivity. Mechanistic studies revealed that LANA synergized with RSL-3 to downregulate GPX4. Furthermore, LANA suppressed Nrf2 expression and impaired its nuclear translocation, while upregulating MDM2 expression. Pharmacological inhibition experiments demonstrated that Nrf2 inhibitors abrogated the ferroptosis-rescuing effects of LANA knockdown, whereas MDM2 inhibitors counteracted the pro-ferroptotic activity of LANA overexpression. These findings collectively established that LANA promotes ferroptosis via dual modulation of Nrf2 signaling and MDM2 upregulation.

## 2. Materials and Methods

### 2.1. Cell Lines, Plasmids, and Chemicals

HeLa cells were maintained in Dulbecco’s modified Eagle’s medium (DMEM; Gibco) supplemented with 10% fetal bovine serum (FBS), 2 mM glutamine, 1 mM sodium pyruvate, and 1% penicillin/streptomycin at 37 °C under 5% CO_2_. KSHV-latent iSLK.219 cells were cultured in DMEM containing 100 μg/mL G418, 4 μg/mL puromycin, and 100 μg/mL hygromycin. The iSLK.219 cell line is a telomerase-immortalized endothelial cell derived from human dermal microvascular endothelia (HDMEC). It stably harbors recombinant rKSHV.219—a KSHV bacmid engineered with constitutive GFP (EF-1α promoter), lytic-inducible RFP (PAN promoter, activated by RTA), and dual antibiotic resistance (neomycin/kanamycin) that expresses RTA under the control of a doxycycline (DOX)-responsive promoter [13,14].

The LANA expression plasmid pCAGGS-LANA (gift from Prof. Ke Lan) was transfected using Lipofectamine 3000 (Invitrogen).

The following ferroptosis inducer and inhibitors were procured from GlpBio: RSL-3 (GPX4 inhibitor, Cat# GC12431), ML385 (Nrf2 inhibitor, Cat# GC19254), and nutlin3a (MDM2 inhibitor, Cat# GC10470).

### 2.2. RNAi Assay

LANA-specific siRNA (si-LANA: 5′-GCATTTGTGTCTAGTCCTA-3′) or nonspecific siRNAs (Riobo, Guangzhou, China) as controls were transfected into iSLK.219 cells using Lipofectamine 3000 (Invitrogen, Carlsbad, CA, USA). Knockdown efficiency was validated using qRT-PCR and western blot.

### 2.3. Cell Viability Assay

Cell viability was assessed using a CCK-8 kit (Beyotime, Shanghai, China) according to the manufacturer’s protocol. Absorbance at 450 nm was measured using a SPARK microplate reader (Tecan, Männedorf, Switzerland). Triplicate experiments were performed for each condition.

### 2.4. Quantitative Real-Time PCR (qPCR)

Total RNA was extracted using TRIzol (Invitrogen, Carlsbad, CA, USA), reverse-transcribed with the Thermoscript™ RT-PCR System, and amplified using SYBR Green Master Mix (Takara Bio Inc., Otsu, Japan) on a QuantStudio 5 Real-Time PCR System (Applied Biosystems, Foster City, USA). Primer sequences are listed in Table 1. Data were normalized to β-actin/GAPDH and analyzed using the 2^−ΔΔCt^ method.

### 2.5. Western Blot

Cells were lysed in RIPA buffer containing protease inhibitors (HALT™, Thermo Fisher, Waltham, MA, USA). Proteins (30 μg/lane) were separated by 10% SDS-PAGE, transferred to PVDF membranes, and probed with primary antibodies against LANA (1:1000, MBL, Beijing, China), GPX4 (1:1000, ABclonal, Woburn, MA, USA), Nrf2 (1:1000, ABclonal), MDM2 (1:1000, ABclonal), and β-actin (1:5000, Abcam, Waltham, MA, USA). Blots were developed using ECL substrate (Millipore, Burlington, MA, USA).

### 2.6. Measurement of Reactive Oxygen Species (ROS) and Malondialdehyde (MDA) Levels

The levels of reactive oxygen species (ROS) were quantified using a ROS assay kit (Beyotime, Shanghai, China. Cat# S0033M) in accordance with the manufacturer’s guidelines. Cells were plated in 6-well plates and subjected to the specified treatments. Following treatment, the cells were washed twice with phosphate-buffered saline (PBS) and subsequently incubated with the ROS detection reagent, dichlorodihydrofluorescein diacetate (DCFH-DA, 5 µmol/L), at 37 °C for 30 min in the dark. After incubation, the cells were washed three times with PBS to remove any unbound reagent. Finally, images were captured using an inverted fluorescence microscope, and the intensity of the immunofluorescence was quantified using ImageJ software (version 1.52).

Lipid peroxidation levels were determined using lipid peroxidation MDA assay kits (Beyotime, Shanghai, China. Cat# S0131M) according to the manufacturer’s instructions. The MDA assay quantifies lipid peroxidation by reacting malondialdehyde (MDA) with thiobarbituric acid (TBA). HeLa or iSLK.219 cells are lysed using RIPA buffer and centrifuged, and supernatants are mixed with TBA working solution. After boiling, samples are centrifuged and absorbance at 532 nm is measured. A standard curve (1–50 μM MDA) is prepared for quantification. Protein concentrations are determined using a BCA assay (Beyotime, Shanghai, China. Cat# P0009) to normalize MDA levels as μmol/mg protein.

### 2.7. Immunofluorescence

Cells grown on glass-bottom plates (Corning) were fixed with 4% paraformaldehyde, permeabilized with 0.1% Triton X-100, and blocked with 1% BSA. After overnight incubation with anti-Nrf2 (1:200, ABclonal) at 4 °C, cells were stained with rhodamine-conjugated secondary antibody (1:200, ZSGB-bio, Beijing, China) and DAPI. Images were captured using a confocal microscope (Olympus, Tokyo, Japan).

### 2.8. Statistical Analysis

Data are presented as mean ± SEM from ≥3 independent experiments. Statistical significance was determined using unpaired two-tailed Student’s *t*-tests, one-way ANOVA, or two-way ANOVA, as implemented in GraphPad Prism (version 9.0). Significance levels are denoted as follows: *, *p* < 0.05; **, *p* < 0.01; ***, *p* < 0.001; ****, *p* < 0.0001; ns, nonsignificant.

## 3. Results

### 3.1. LANA Knockdown Attenuates Ferroptosis Sensitivity in KSHV-Infected Cells

**Figure 1:**
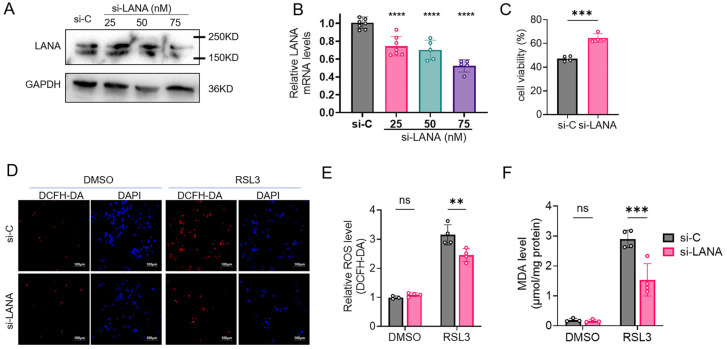
Knocking down LANA suppresses ferroptosis in iSLK.219 cells. (**A**,**B**) Nonspecific siRNA (si-C) or si-LANA was transfected into iSLK.219 cells. The cells were harvested 24 h after siRNA transfection, and the protein and mRNA levels were determined using western blot and qPCR assays. (**C**) iSLK.219 cells were exposed to 15μM RSL-3 24 h after siRNA transfection and incubated for the following 24 h. The viability of the cells was then tested using a CCK-8 assay. (**D**) Total ROS levels were detected using a ROS kit, and images (ROS probe (red); DAPI-stained nuclei (blue))were taken at 10 × 20 magnification using a fluorescence microscope (scale bar 100 μm). (**E**) Quantitative plot of the fluorescence intensity for each group. (**F**) The lipid peroxidation levels were assayed using a MDA kit, and the protein content was assayed via a BCA kit; the MDA content was labeled in μmol/mg protein. Significance was assessed through using one-way ANOVA (**B**,**C**) or two way ANOVA (**E**,**F**) with *p* values and represented as follows: **, *p* < 0.01; ***, *p* < 0.001; ****, *p* < 0.0001; ns, nonsignificant.

KSHV-latent iSLK.219 cells were transfected with LANA-specific siRNA (si-LANA) or scrambled control (si-C). The cells were harvested, and western blot and qPCR confirmed efficient LANA silencing (Figure 1A,B). Twenty-four hours after transfection with 75 nM si-LANA, both the mRNA and protein levels of LANA in cells were decreased by about 50%. Cells treated with 15 μM RSL-3 for 24 h exhibited reduced cell death upon LANA knockdown. si-LANA significantly reduce RSL-3 induced cell death (Figure 1C). Although basal ROS and MDA levels remained unchanged in si-LANA cells, ferroptosis inducer treatment significantly reduced ROS accumulation and MDA production, with maximal attenuation observed in RSL-3-challenged cells (Figure 1E,F).

### 3.2. LANA Overexpression Sensitizes HeLa Cells to Ferroptosis

**Figure 2:**
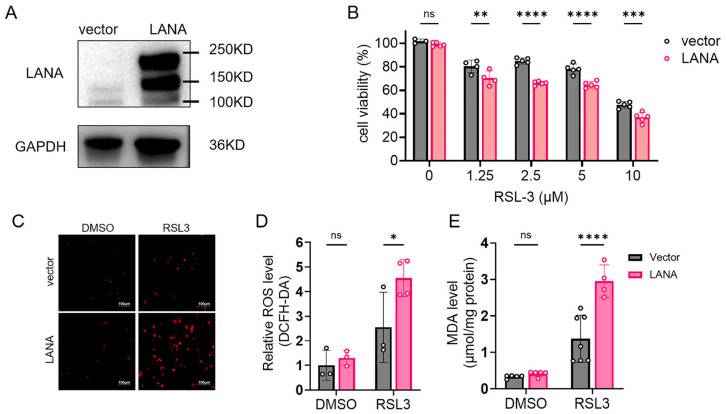
Overexpression of LANA enhances ferroptosis in HeLa cells. (**A**) HeLa cells were transfected with pCAGGS-LANA or empty vector, and the protein levels of LANA were assessed with western blot analysis. (**B**) After 24 h of transfection, the cells were treated with various doses of RSL-3 for 24 h, and the viability of the cells was measured using a CCK-8 assay. (**C**) Total ROS levels were detected using a ROS kit, and images (ROS probe (red)) were taken at 10 × 20 magnification with a fluorescence microscope (scale bar 100 μm). (**D**) Quantitative plot of the fluorescence intensity for each group. (**E**) Lipid peroxidation levels were assayed using a MDA kit, and protein content was assayed using a BCA kit to determine the MDA content in μmol/mg protein. Significance was assessed with two-way ANOVA with *p* values and represented as follows: *, *p* < 0.05; **, *p* < 0.01; ***, *p* < 0.001; ****, *p* < 0.0001; ns, nonsignificant.

To further investigate LANA’s role in ferroptosis, HeLa cells were transfected with either the pCAGGS-LANA plasmid or empty vector control (pCAGGS). Western blot analysis confirmed successful LANA overexpression in transfected cells (Figure 2A). Twenty-four hours post-transfection, cells were exposed to serials concentration of RSL3 for an additional 24 h prior to viability assessment. Notably, LANA-overexpressing cells exhibited significantly reduced viability compared to vector controls when treated with RSL-3 (Figure 2B). Although basal levels of ROS and MDA remained unaltered by LANA overexpression in isolation, cells expressing LANA and treated with RSL-3 exhibited elevations in both ROS accumulation and MDA production (Figure 2C–E).

### 3.3. LANA Represses GPX4 Expression

**Figure 3:**
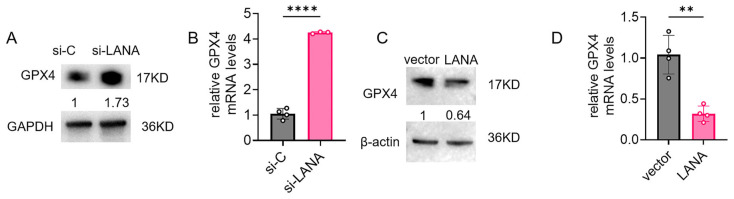
LANA suppressed the expression of ferroptosis-resistance genes. (**A**,**B**) Nonspecific siRNA (si-C) or si-LANA was transfected into iSLK.219 cells. The cells were harvested 24 h after siRNA transfection, and the GPX4 protein and mRNA levels were determined using western blot and qPCR assays. (**C**,**D**) HeLa cells were transfected with pCAGGS-LANA or empty vector, and the protein and mRNA levels of GPX4 were assessed using western blot and qPCR assays. Statistical significance was assessed by unpaired *t*-test, with *p*-values denoted as follows: **, *p* < 0.01; ****, *p* < 0.0001; ns, nonsignificant.

Mechanistic investigations revealed that LANA knockdown upregulated GPX4 expression levels (Figure 3A,B). Conversely, LANA overexpression suppressed GPX4 expression (Figure 3C,D). These results suggest its central role in LANA-mediated ferroptosis potentiation.

### 3.4. LANA Modulates Nrf2 Suppression

**Figure 4:**
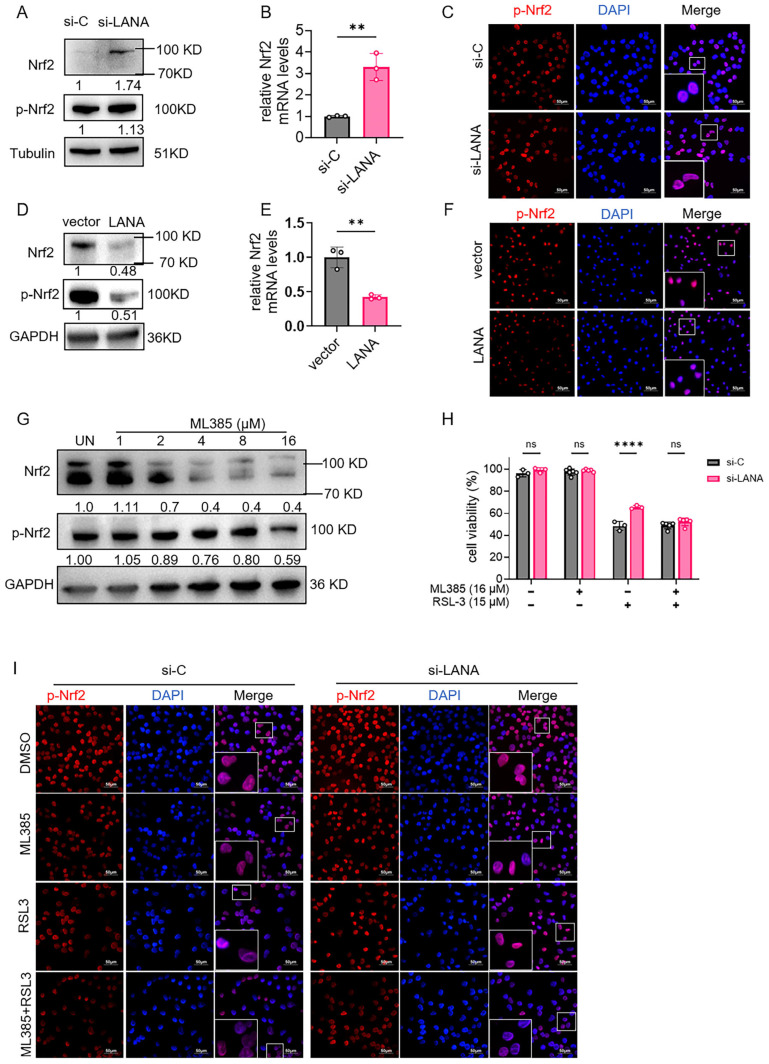
Knocking down of LANA suppresses ferroptosis by activating Nrf2 activity. (**A**–**C**) Nonspecific siRNA (si-C) or si-LANA was transfected into iSLK.219 cells. The cells were harvested 24 h after siRNA transfection, and Nrf2 and p-Nrf2 protein levels were determined using western blot assays. Relative Nrf2 mRNA levels were determined using qPCR assays, and nuclear localization was determined using western blot and IF assays. (**D**–**F**) HeLa cells were transfected with pCAGGS-LANA or empty vector, and Nrf2 and p-Nrf2 protein levels were determined using western blot assay. Relative Nrf2 mRNA levels were determined using qPCR assays, and nuclear localization was determined using western blot and IF assays. (**G**) iSLK.219 cells were exposed to different concentrations of the Nrf2 inhibitor ML385 and incubated for 24 h. The protein expression levels of Nrf2 and p-Nrf2 were assayed with western blots. (**H**,**I**) Nonspecific siRNA (si-C) or si-LANA was transfected into iSLK.219 cells, which were then exposed to 15 μM RSL3 and/or 16 μM ML385. The viability of the cells and nuclear localization were then tested using CCK-8 and IF assays. Scale bar 50 μm. pNrf2 was stained red; nuclei were counterstained with DAPI (blue), and, high-magnification insets demonstrate the co-localization of pNrf2 with nuclei (**C**,**F**,**H**). Statistical significance was assessed using unpaired *t*-test (**B**,**E**) or two-way ANOVA (**H**) with *p*-values denoted as follows: **, *p* < 0.01; ****, *p*< 0.0001; ns, nonsignificant.

In iSLK.219 cells, the knockdown of LANA led to a significant increase in the total level of Nrf2 (by 1.74-fold), while the phosphorylated form of Nrf2 exhibited only a mild increase (Figure 4A). The knockdown of LANA enhanced the nuclear translocation of Nrf2 (Figure 4B). LANA overexpression in HeLa cells reduced Nrf2 and p-Nrf2 (Figure 4C) and impaired nuclear accumulation (Figure 4D). ML385, an Nrf2 inhibitor, decreased total Nrf2 and p-Nrf2 in a dose-dependent manner (Figure 4E). Immunofluorescence assays demonstrated that ML385 mitigated the reduction in nuclear phosphorylated Nrf2 levels induced by LANA knockdown (Figure 4G). These results indicate that Nrf2 is involved in rescuing ferroptosis by silencing LANA.

### 3.5. LANA Upregulates MDM2 Expression

**Figure 5:**
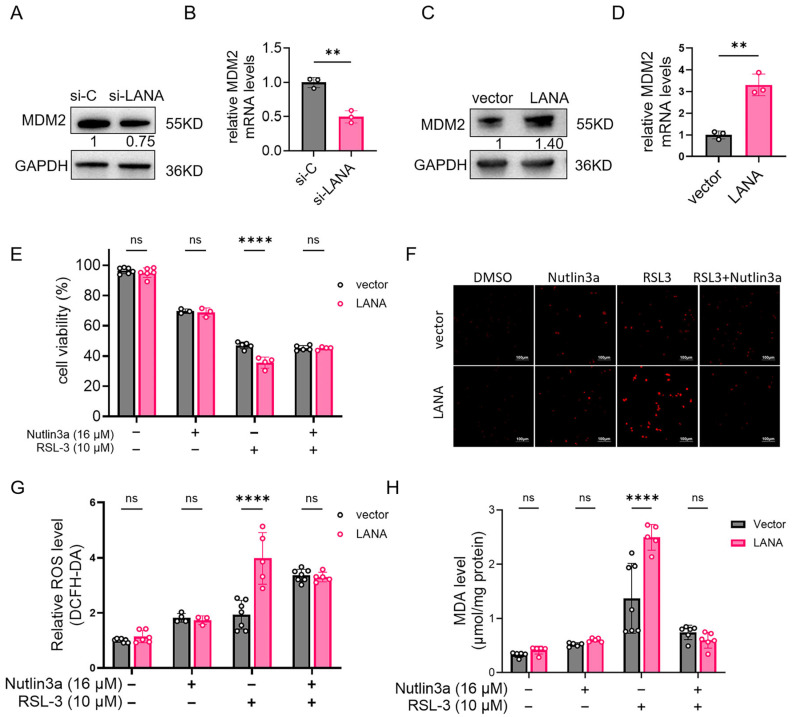
LANA overexpression enhances ferroptosis by upregulating MDM2. (**A**,**B**) Nonspecific siRNA (si-C) or si-LANA was transfected into iSLK.219 cells. The cells were harvested 24 h after siRNA transfection, and MDM2 protein and the mRNA levels were determined using western blot and qPCR. (**C**,**D**) HeLa cells were transfected with pCAGGS-LANA or empty vector, and MDM2 protein and the mRNA levels were determined using western blot and qPCR. (**E**) Exposure to 10 μM RSL3 with or without 16 μM nutlin3a and detection of cell viability using a CCK-8 assay. (**F**) Total ROS levels were detected using a ROS kit, and images (ROS probe (red)) were taken at 10 × 20 magnification with a fluorescence microscope (scale bar 100 μm). (**G**) Quantitative plot of the fluorescence intensity for each group. (**H**) Lipid peroxidation levels were assayed using a MDA kit, and protein content was assayed using a BCA kit; the MDA content was labeled in μmol/mg protein. Statistical significance was assessed with unpaired *t*-test (**B**,**D**), or two-way ANOVA (**G**,**H**) with *p*-values denoted as follows **, *p* < 0.01; ****, *p* < 0.0001; ns, nonsignificant.

LANA knockdown decreased MDM2 protein levels in iSLK.219 cells, while overexpression increased MDM2 in HeLa cells (Figure 5A,B). Co-treatment with the MDM2 inhibitor nutlin3a (10 μM) abrogated LANA-enhanced ferroptotic cell death (Figure 5C) and attenuated ROS/MDA elevation (Figure 5D–F). Notably, nutlin3a completely neutralized the differential effects between LANA-expressing and control cells (*p* > 0.05).

## 4. Discussion

As a multifunctional regulator encoded by KSHV ORF73 [6,15], LANA has been established as a potent inhibitor of apoptosis in previous investigations [9,11]. Notably, LANA expression levels increased during the lytic replication phase compared to the latent phase, despite its characterization as a hallmark latent protein [16]. Notably, recent genetic evidence demonstrates that LANA-null KSHV mutants exhibit complete abrogation of lytic replication capacity, establishing its indispensable role in both viral life cycle phases [17]. Emerging studies by Zhou et al. further identify that KSHV infection confers cellular resistance to ferroptosis—an iron-dependent regulated cell death modality—through undefined mechanisms [18]. The latent viral proteome, comprising LANA, vCyclin (ORF72), vFLIP (ORF71), and viral miRNAs, collectively orchestrates complex pathogenic cascades. According to the research reported by Ma Q et al., the expression of the KSHV major latent transcript (LAT), which encompasses LANA, v-cyclin, and vFLIP, in 293 cells elevated ROS production [19]. Nevertheless, current research remains inconclusive regarding the specific role of LANA in ferroptosis regulation.

Comparative analysis with Epstein-Barr virus (EBV), another γ-herpesvirus, reveals distinct ferroptosis regulatory strategies. EBV latency proteins activate the p62–Keap1–Nrf2 axis to transcriptionally upregulate SLC7A11 and GPX4 expression, thereby establishing ferroptosis resistance [20]. Mechanistically, EBNA1-mediated Nrf2 stabilization proves essential for maintaining GPX4 levels and counteracting ferroptotic stress [21]. These findings prompt critical questions regarding potential functional conservation between EBV and KSHV latency proteins in ferroptosis regulation. Although preliminary evidence suggests that KSHV infection broadly inhibits ferroptosis, the specific role of LANA requires careful differentiation from other latent proteins.

Importantly, molecular regulators of apoptosis and ferroptosis frequently exhibit functional divergence—exemplified by MDM2, which simultaneously suppresses apoptosis while promoting ferroptosis [10]. This functional dichotomy underscores the necessity for precise molecular dissection of LANA’s role in cell death regulation. Compounding this complexity, recent studies paradoxically identify LANA as a bifunctional determinant essential for both latent persistence and lytic reactivation [17]. These emerging insights highlight the multifaceted nature of LANA’s biological activities, necessitating systematic investigation of its ferroptosis-related functions.

Our experimental system employed complementary gain- and loss-of-function approaches to delineate LANA’s ferroptosis regulatory effects. Unexpectedly, the results of the present study indicate that LANA enhances sensitivity to ferroptosis. iSLK.219 cells with LANA knockdown or HeLa cells overexpressing LANA alone showed limited effects on cell viability within 24–48 h. However, iSLK.219 cells transfected with si-LANA were found to be less sensitive to RSL-3-induced ferroptosis than those transfected with nonspecific siRNA. Conversely, HeLa cells transfected with LANA exhibited increased sensitivity to ferroptosis compared with those transfected with empty vectors. Altering LANA expression alone did not affect the basal ROS or MDA content. However, when exposed to RSL-3, iSLK.219 cells with LANA knockdown presented lower levels of ROS and MDA, whereas HeLa cells overexpressing LANA presented higher levels of ROS and MDA.

Mechanistically, LANA modulated key ferroptosis regulators. LANA downregulates the expression of several ferroptosis-related genes, especially GPX4. In addition, LANA promotes ferroptosis by downregulating Nrf2 protein levels, reducing its phosphorylation levels and nuclear localization, and increasing MDM2 expression. Ferroptosis is regulated by various genes, such as GPX4 and Nrf2. Therefore, we examined the effects of LANA on the expression of these genes. The results showed that the knockdown of LANA in iSLK.219 cells led to the upregulation of GPX4, whereas the overexpression of LANA in HeLa cells resulted in the downregulation of GPX4. Nrf2, a transcription factor, regulates the expression of various genes associated with ferroptosis. Its role involves inhibiting cellular iron uptake and limiting ROS production [22,23]. The Nrf2 inhibitor ML385 increases the sensitivity of acute myeloid leukemia cells to RSL3-induced ferroptosis [24]. Nrf2 plays a crucial role in KSHV pathogenesis and replication regulation. KSHV-positive murine KS-like tumor cells exhibit elevated levels of Nrf2 nuclear localization following phosphorylation [25]. Nrf2 is activated upon KSHV infection [26] and colocalizes with LANA in the nucleus [15]. MDM2 plays an important role in the regulation of KSHV replication and inhibits RTA expression [27,28,29,30]. Venkatesh and colleagues reported that MDM2 and MDMX promote ferroptosis independently of p53 [10]. The MDM2 inhibitor nutlin3a has been shown to promote apoptosis in KSHV-positive cells [31]; however, it also inhibits ferroptosis mediated by MDM2 [10]. However, the impact of LANA on the expression of Nrf2 and MDM2 are still unclear. In this study, these results indicate that LANA regulates ferroptosis partly through downregulating Nrf2 and upregulating MDM2.

These results unexpectedly demonstrated that LANA can promote ferroptosis. This finding intriguingly contradicts prior reports indicating that KSHV infection broadly increases cellular resistance to ferroptosis [18]. This apparent contradiction suggests that LANA itself is not the primary viral factor responsible for such resistance. Instead, other viral components likely compensate, particularly during latency—a phase where infected cells maintain elevated iron levels yet resist ferroptosis. LANA expression is significantly higher during lytic replication compared to latency. Reactive oxygen species (ROS) are known activators of KSHV lytic replication. Elevated LANA levels during lytic replication might synergistically enhance ROS production. Increased ROS levels directly facilitate the transition to or progression of the lytic cycle and simultaneously sensitize the cell to ferroptosis. LANA-mediated ferroptosis sensitization represents a lytic-phase adaptation by promoting ROS-driven viral reactivation.

Notably, our study has limitations. First, the experiments were conducted in vitro; validating these findings in animal models is critical. Second, the interplay between LANA and other KSHV latent proteins (e.g., vFLIP) in ferroptosis regulation warrants further exploration. Lastly, HeLa cells are non-ideal for modeling KSHV infection. While HeLa cells lack native KSHV tropism, their E6/E7-mediated p53/Rb suppression [32] partially recapitulates KSHV-induced tumor suppressor inactivation, providing mechanistic insights. HPV^+^ cancers exhibit Nrf2 downregulation and ROS elevation [32], paralleling our LANA overexpression phenotypes.

Despite these limitations, this work establishes LANA as a key modulator of ferroptosis via GPX4 suppression, Nrf2 inhibition, and MDM2 upregulation. These findings offer new insights into KSHV pathogenesis and potential therapeutic strategies for KSHV-associated malignancies.

## 5. Conclusions

This study reveals that KSHV-encoded LANA promotes iron-dependent ferroptosis by suppressing Nrf2, reducing GPX4 expression, and upregulating MDM2, contrasting with KSHV’s general ferroptosis resistance. These findings highlight LANA’s dual regulatory role, challenge existing paradigms of viral modulation of cell death, and suggest potential therapeutic strategies targeting ferroptosis pathways in KSHV-associated malignancies.

## Figures and Tables

**Table 1:** Primer sequences for quantitative real-time PCR.

Genes	Primer Sequences (5′-3′)
LANA	For: GCCTACATCTCCCATCTCCA
	Rev: ATCCTCCTCGTCATCCTCCT
GPX4	For: ACACCGTCTCTCCACAGTTC
	Rev: ACGCTGGATTTTCGGGTCTG
MDM2	For: TGTTTGGCGTGCCAAGCTTCTC
	Rev: CACAGATGTACCTGAGTCCGATG
Nrf2	For: AGCACATCCAGTCAGAAACCAGT
	Rev: GGAATGTCTGCGCCAAAAGCTG
β-actin	For: GGGAAATCGTGCGTGACAT
	Rev: GTCAGGCAGCTCGTAGCTCTT

## Data Availability

The data used in this study are available from the corresponding author upon reasonable request.
